# The Evolution of Machine Learning in Medicinal Chemistry: A Comprehensive Bibliometric Analysis

**DOI:** 10.2174/011570159X384988250430093924

**Published:** 2025-05-13

**Authors:** Yanhua Wang, Tongxin Guan, Dongyu Xu, Mingyan Liu, Zhichang Zhang

**Affiliations:** 1 Department of Computer, School of Intelligent Medicine, China Medical University, Shenyang, China;; 2 Innovation Institute, China Medical University, Shenyang, China;; 3 Department of Pharmacology, School of Pharmacy, China Medical University, Shenyang, China

**Keywords:** Artificial intelligence, bibliometrics, deep learning, machine learning, medicinal chemistry, drug discovery

## Abstract

**Introduction:**

In the medicinal chemistry (MC) field, artificial intelligence (AI) has been used to establish quantitative structure-activity relationship (QSAR) classification models, virtual screening, drug discovery, drug design, and so on. In this investigation, MC AI studies (AI-MC) (from 2001-2023) underwent quantitative and qualitative modeling analyses.

**Methods:**

Using a hybrid research strategy incorporating content analyses and bibliometric methods, we retrospectively analysed the AI-MC literature using a bibliometrix package (R software) combined with CiteSpace V and VOSviewer programs.

**Results:**

Between 2001 and 2023, AI-MC articles were published in 92 countries or regions, with China and the United States leading in the number of publications. Also, 196 affiliations were added to AI-MC research; the CHINESE ACADEMY OF SCIENCES contributed the most. Reference clusters were categorized as follows: (1) QSAR, (2) virtual screening, (3) drug discovery, (4) drug design. Predictive model (2020-2021), molecular fingerprints (2021-2023) and scoring function (2021-2023) reflected research frontier keywords. As we look to the future, the ongoing progress and innovation in technology herald the promising development of multimodal and large language models (LLMs) within the realm of MC.

**Discussion:**

The integration of AI into MC has significantly transformed the landscape of drug development. AI techniques, particularly machine learning, and deep learning algorithms, have demonstrated remarkable potential in accelerating the discovery and optimization of new drugs. By leveraging large datasets and advanced computational models, AI enhances the efficiency of virtual screening, improves the accuracy of QSAR models, and facilitates the design of novel therapeutic agents. As the technology continues to advance, the development of multimodal and large language models (LLMs) is expected to further revolutionize this field, offering new opportunities for more precise and efficient drug design and discovery.

**Conclusion:**

We comprehensively characterized the AI-MC field and determined future trends and hotspots. Importantly, we provided a dynamic oversight of the AI-MC literature and identified key upcoming research areas.

## 1 INTRODUCTION

Medicinal chemistry (MC) primarily studies the interaction between the molecular structures of different compounds and potential therapeutic targets for diseases, mainly involving the design, discovery, synthesis, identification, and interpretation of bioactive compounds at the molecular level [1]. MC contributes to all facets of biologically active molecule/compound discovery, with a view to unraveling metabolism, interpreting the mode of actions at molecular levels, and generating structure-activity relationships [2]. MC not only plays an important role in the field of small molecule drug discovery [3] but also drives the research and development process of coupled drugs [4].

In the 21^st^ century, artificial intelligence (AI) has become one of the most disruptive societal technologies, with great transformative potential [5]. Common AI methods include incorporating classical support-vector machine (SVM) and neural networks, machine learning (ML) for structured data, modern deep learning (DL), and natural language processing of unstructured data [6]. Artificial intelligence large-scale models can process massive amounts of data and complete various complex tasks [7]. AI is important in analyzing and systemizing large datasets using statistical ML strategies.

Machine learning (ML), a cornerstone of modern AI, employs mathematical and statistical algorithms to identify complex patterns in data and predict variables of interest. This approach has revolutionized clinical decision-making and drug development [8-10]. Unlike traditional statistical methods, ML models like decision trees can process binary or categorical data, enabling the derivation of novel variables and structured classification of medical information [11, 12]. Advanced AI-based ML approaches exert significant impacts on DD processes in MC [13]. Increasingly, MC scientists are exploiting different AI techniques to comprehensively evaluate and predict the biological effects of different molecules and chemicals [14]. AI has significantly impacted MC, particularly in building quantitative structure-activity relationship (QSAR) classification models, virtual screening, drug discovery, and drug design. To promote AI-MC research and development, bibliometrics and studies can provide researchers/scientists with invaluable insights into its evolution and future prospects.

We used a database search strategy (January 1, 2001-December 31, 2023) and bibliometrics to examine the AI-MC literature, incorporating studies from different areas. We investigated journals publishing AI-MC research, analyzed the top ten cited references and enumerated how many times popular references were cited. After clustering reference networks related to co-cited references, we examined the AI-MC knowledge base. We also classified AI-MC research into clusters, performed comprehensive content analyses, identified future research pathways, and provided a macro-understanding and micro-analysis of AI-MC research. In comparison to conventional systematic reviews, our logical framework tracked AI-MC developments and identified unique knowledge areas over time.

## 2 MATERIALS AND METHODS

### 2.1 Data Sources and Search Strategies

In June 2024, data from the Web of Science Core Collection were selected, with search terms covering the period from 2001 to 2023, and were independently verified by three study authors. Search terms were: TS= (“machine learning” OR “decision tree” OR “random forest” OR “logistics regression” OR “support vector machine” OR “Naive Bayes model” OR “k nearest neighbours” OR “k-means” OR “Adaboost” OR “Markov chain” OR “Deep Learning” OR “Convolutional Neural Network*” OR “Recurrent Neural Network*” OR “Fully Convolutional Network*” OR “Generative Adversarial Network” OR “Reinforcement Learning” OR “Back Propagation” OR “Fully Neural Network” OR “Recursive Neural Network” OR “Autoencoder” OR “Deep Belief Network” OR “Restricted Boltzmann machine” OR “Transformers” OR “Graph Convolution Networks” OR “graph neural network” OR “deep residual network” OR “ResNet” OR “variational autoencoder” OR “adversarial autoencoder” OR “message passing neural network”). The category was limited to medicinal chemistry and the document types were restricted to articles or reviews. Basic information included number of papers/citations, year of publication, keywords, countries/regions, affiliations, journals, and references. Exclusion criteria: (1) non-pertinent meeting abstracts/proceedings papers, book chapters, data papers, editorials, and repeated articles, and (2) unpublished studies with limited data. The screening process is illustrated in Fig. (**1**). Finally, 2751 AI-MC publications were obtained, ensuring the stability requirements of bibliometric analysis.

### 2.2 Bibliometric Analyses

The bibliometric analyses were performed using VOSviewer, R, and CiteSpace. Bibliometrics is a powerful quantitative approach that examines bibliographic materials; it processes knowledge domains, identifies cognitive-epistemological structures, and classifies data from many variables, *e.g*., institutions, journals, and countries [15, 16].

We incorporated most publication traits, including institutes, journals, countries, and keywords. For co-occurrence analyses and collaborative network visualization of traits, we used the Online Analysis Platform of Literature Metrology, CiteSpace V (Drexel University, USA), and VOSviewer (Leiden University, Holland).

In CiteSpace, we conducted reference co-citation analyses, constructed knowledge maps, and determined burst keywords (BKs) to identify novel recurring keywords. In R, statistical analyses and graphic displays were integrated. We then performed basic bibliometric analyses on cleansed data and local citation score (LCS) in the bibliometrix package in R [17].

## 3 RESULTS

### 3.1 Publication Summary

**Fig. (2):**
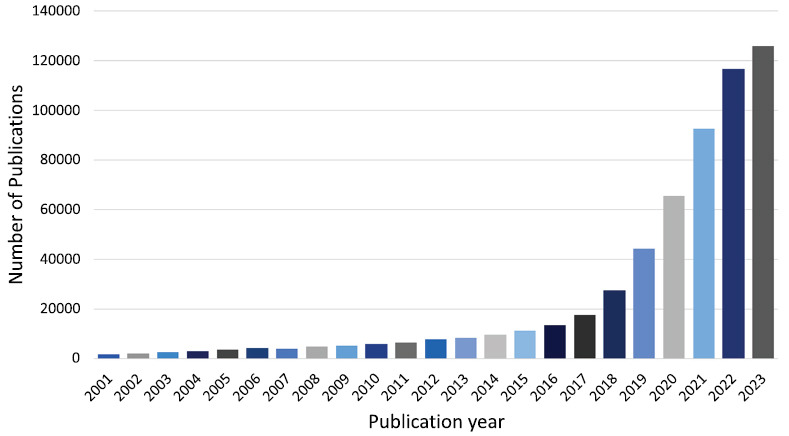
Trends in the number of publications on AI-MC from 2001 to 2023.

Between 2001 and 2023, 2751 AI-MC studies were published; 65,938 references and 3,688 keywords were recorded, and several AI-MC trends were identified (Fig. **2**). Publication numbers increased year on year and suggested an important research trend had been established.

### 3.2 Country/Affiliation Analyses

**Fig. (3):**
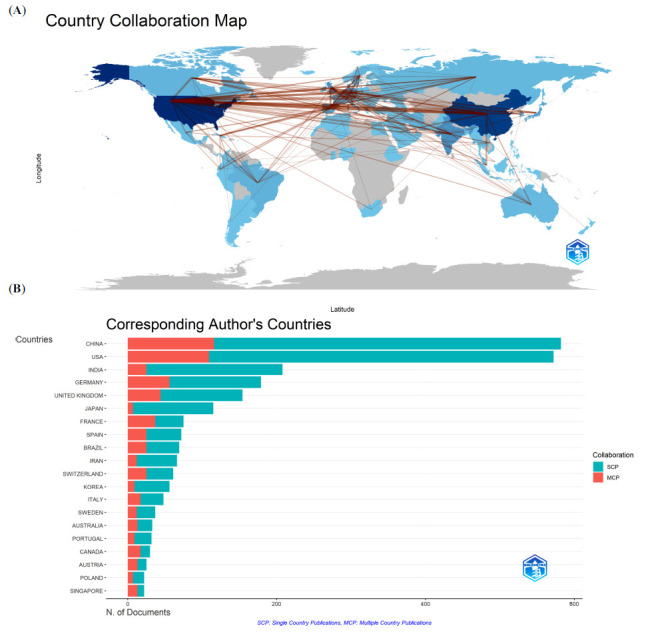
The cooperation of countries/regions contributed to publications. (**A**) Country Collaboration map. (**B**) Most productive countries.

Publications came from 92 countries; the top ten are shown (Table **1**). Using the bibliometrix package, these countries were distributed across Europe, North America, Asia, and South America, but primarily Asia (n = 4) and Europe (n = 4). China published the most studies (582, 21.2%), then the USA (572, 20.8%). China and the United States collectively accounted for 42% of all publications. Next, a collaborative network was established using publication numbers and relationships in countries (Fig. **3A**). The USA and China displayed high centrality values (dark blue, Fig. **3A**), which suggested important roles and significant contributions to AI-MC research. Importantly, considerable active cooperation was identified between countries. As indicated (Fig. **3A**), thicker red lines showed closer cooperation, *e.g*., the USA cooperated closely with the UK, China, Canada, and Germany, while China cooperated actively with the UK, Australia, and France. In terms of the largest number of single- and multi-country publications, China had the highest, followed by the USA (Fig. **3B**).

In total, 196 affiliations contributed to AI-MC research; the top ten are shown (Table **1**). The Chinese Academy of Sciences had the most publications (75), then Centre National de la Recherche Scientifique CNRS (66) and the University of California System (62).

### 3.3 Higher-impact Journal and Article Analyses

Generally, referential relationships between academic journals reflect knowledge exchange in a research field, where citing studies represent knowledge frontiers and cited studies the knowledge base. The Journal of Chemical Information And Modelling published the most AI-MC studies (612), followed by Molecular Informatics (142) and Current Topics In Medicinal Chemistry (67) (Table **2**).

**Fig. (4):**
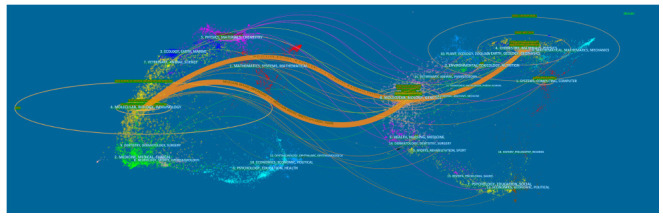
The dual-map overlay of journals contributed to publications on AI-MC from 2001 to 2023.

An overlay dual-map of journals (Fig. **4**) shows citing journals (left), cited journals (right), and citation relationships (colored lines). The lines showed that studies in Material Physics/Chemistry journals were typically cited in studies in Molecular Biology/Immunology journals.

**Fig. (5):**
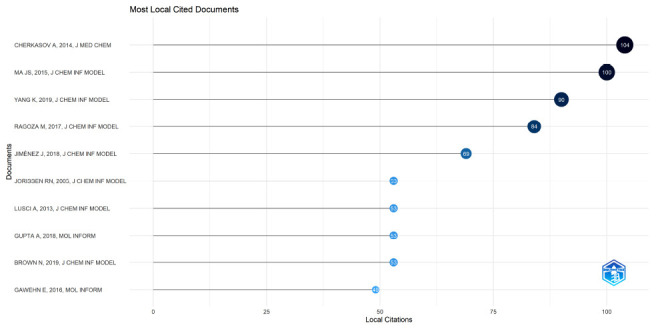
The number of citations and the top ten highly cited documents in this field from 2001 to 2023.

Citation analyses are used to investigate highly cited articles; citation frequencies can often reflect a study’s influence in a particular research area [18]. Also, referential relationships between citing investigations reflect knowledge frontiers, and referenced investigations reflect the knowledge base. As outlined (Fig. **5** and Table **3**), the ten highly cited AI-MC articles were generated between 2005 and 2019 and focused on drug research modeling, virtual screening, drug design, and drug discovery [19-29].

### 3.4 Research Hotspot Analyses

In recent years, the AI-MC field has been relatively successful and generated significant research output. We used VOSviewer to extract and classify keywords from the dataset, revealing research directions, and exploring and discovering research hotspots in the AI-MC field. The co-occurrence network includes all keywords with a connection strength of no less than 16, which are divided into four clusters with different colors. Cluster 1 is red and describes the application of AI in drug modeling. Cluster 2 is green and describes the application of AI in virtual screening. Cluster 3 is yellow and describes the application of AI in drug discovery. Cluster 4 is blue and describes the application of AI in drug design.

**Fig. (6):**
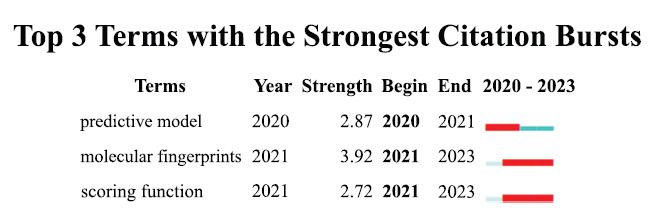
Top 3 terms with the strongest citation bursts.

Keywords distill research into contemporary concepts/ research topics, while BKs reflect research frontiers and emerging trends. In CiteSpace, we captured predictive model(2020-2021), molecular fingerprints (2021-2023), and scoring function (2021-2023) BKs (Fig. **6**) which possibly represent current MC hotspots.

## 4 DISCUSSION

### 4.1 Research Clusters

**Fig. (7):**
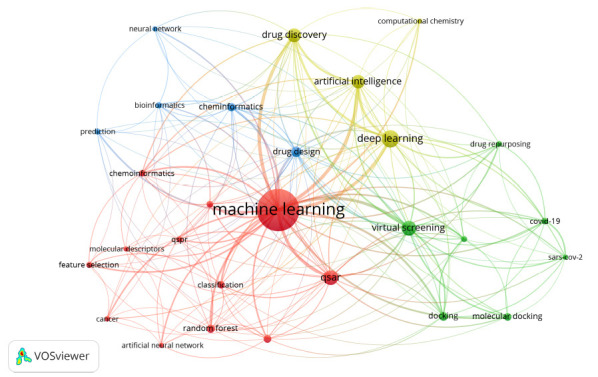
Visualization of keyword co-occurrence and cluster networks.

**Fig. (8):**
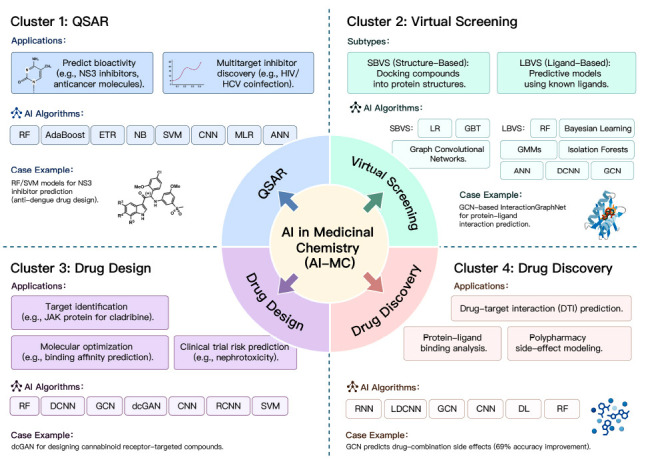
Mechanistic map of AI-MC research: A four-cluster framework integrating algorithms, applications, and case example.

We constructed a network visualization of co-occurring author keywords to determine the main research areas of AI in MC (Fig. **7**). The research field is divided into the following four directions: the application of AI in drug modeling, virtual screening, drug discovery, and drug design (Table **4**). To systematically visualize the interdisciplinary convergence of AI and MC, we developed a four-cluster mechanistic framework (Fig. **8**) that maps cutting-edge algorithms to their therapeutic applications.

#### 4.1.1 Cluster 1: Drug Modeling Research Using AI

This cluster includes the main keyword QSAR. The full name of QSAR is quantitative structure-activity relationship, which is frequently used in modern-day drug design and other related research domains [30]. QSAR is one of the most important strategies for successfully designing new molecules [31]. QSAR has been successfully used for modeling in areas such as prediction and search for inhibitors and prediction and recognition of anticancer molecular activity. Creating QSAR models using AI methods can accelerate the drug development process and save a lot of time and effort [32].

QSAR is a pivotal tool for identifying potent inhibitors, leveraging AI techniques like RF, AdaBoost, ERT, NB, SVM, MLR, CNN, and ANN. Notably, RF, AdaBoost, ERT, and SVM-based models have accurately predicted the efficacy of NS3 and Tallinn inhibitors, aiding in the development of treatments for dengue and Alzheimer's diseases [33]. Explainable Artificial Intelligence (XAI) enhances transparency and interpretability in AI models, which is crucial for trust and accountability in healthcare [34-36]. Kamal *et al*. proposed two explainable artificial intelligence (XAI) methods for interpreting prediction outputs in an effort to boost reliability. Pixel density analysis (PDA) for MRI images and probabilistic graphical modeling (PGM) for genetic data significantly enhance both transparency and reliability in cerebral microbleeds (CMBs) detection and Alzheimer's disease (AD) severity assessment [37]. Excess reactive oxygen species (ROS) damage lipids, proteins, and DNA, leading to neuronal apoptosis and neuroinflammation [38, 39]. AI-driven QSAR models can be instrumental in predicting compounds that modulate the Nrf2 pathway, offering potential therapeutic strategies for Alzheimer's disease and related disorders [40]. Wei *et al*. employed NB and SVM to construct a multi-QSAR model that forecasts chemical-protein interactions, facilitating the discovery of multitarget inhibitors for HIV-1 and HCV coinfection [41]. One-dimensional CNN-based QSAR models have proven effective in identifying anthrax inhibitors and analyzing chemical space, contributing to the discovery of potential anthrax drugs [42]. Worachartcheewan *et al*. utilized MLR, ANN, and SVM to develop a QSAR model for HIV inhibitors, demonstrating robust predictive capabilities and guiding the rational design of novel anti-HIV drugs [43].

AI-driven QSAR models are instrumental in the rapid prediction and identification of anti-cancer molecule activity, accelerating the drug discovery process for cancer treatment [44, 45]. These models employ AI technologies such as RF, SVM, MLR, CNN, and ANN to forecast the activity of anti-cancer molecules and guide drug development. Patricio *et al*. developed a QSAR model utilizing RF, SVM, and CNN to predict cancer protein activity and identify potential anti-cancer molecules, particularly focusing on colorectal cancer (CRC) treatment [46]. Jayaprakash Venkatesan and colleagues constructed a QSAR model with MLR and SVM to uncover anticancer leads [47]. Shayanfar *et al*. created a QSAR model using MLR, ANN, and SVM to predict the activity of Farnesyltranseferase inhibitors (FTIs), with ANN demonstrating higher accuracy [48].

#### 4.1.2 Cluster 2: Virtual Screening Using AI

This cluster includes the main keyword, virtual screening (VS), which is a highly effective drug development method. VS is mainly divided into Structure-based Virtual Screening (SBVS) and Ligand-based Virtual Screening (LBVS) [49]. ML and DL are of great significance in VS. They can solve the problem of expensive and slow drug development, which can be achieved in a cost-effective manner in the shortest possible time through high-quality data collection [50-55].

SBVS is a type of VS based on receptor biomolecular structure that involves docking compounds into target protein structures [56]. AI methods such as logistic regression (LR), gradient boosting trees (GBT), and graph convolutional have played a role in the field of SBVS. Ricci Lopez *et al*. trained two ML classifiers: LR and GBT. It has been demonstrated through experiments that using ML methods on integrated docking results can improve the performance of SBVS. The results of GBT are significantly better than those of LR [57]. Jiang *et al*. developed a deep graph representation learning framework, Interactive Graph Network (IGN), using graph convolutional methods. This framework achieves better SBVS by successfully learning key features of protein-ligand interactions [58].

LBVS (Ligand-Based Virtual Screening) is a valuable technique that generates predictive models for small molecule activity using known ligand information without the need for protein crystal structures [49]. It has been enhanced by AI methods, including RF, Bayesian learning, GMMs, Isolation Forests, ANN, DCNN, and GCN. Shimizu *et al*. performed LBVS on the DLiP PPI library using RF models, identifying 15 novel structured hit compounds [59]. Zheng *et al*. developed an online LBVS platform with a Bayesian learning model, achieving an average AUC of 0.86 and a true hit rate of 73% [60]. Bonanno Etienne *et al*. used GMMs, Isolation Forests, and ANNs to construct an LBVS model, significantly improving LBVS performance by 291% for LECtrained models and 430% for full conformer-trained models, with maximum improvements of 829% and 940% respectively [61]. Berhail *et al*. designed a DCNN-based LBVS model, enhancing the screening process and predicting molecular biological activity effectively [62]. Ani *et al*. employed GCN for LBVS to identify medicinal compounds from plants for hemochromatosis treatment, with GCN outperforming other models in LBVS with a 98.26% accuracy rate [63].

The integration of Ligand-Based Virtual Screening (LBVS) with Structure-Based Virtual Screening (SBVS) can overcome individual limitations, with AI methods like Random Forest (RF) enhancing this synergy. Schneider *et al*. investigated the impact of SBVS and LBVS on affinity prediction using RF, finding that their combination offers high accuracy and robustness, particularly useful for predicting binding affinity and drug profiles [64]. Mubashir *et al*. applied SBVS and LBVS with RF to predict Monoamine Oxidase B (MAO-B) inhibitor activity, aiding in Parkinson's disease (PD) drug development [65]. Wang *et al*. highlighted the benefits of integrating supervised learning into SBVS and LBVS for coronavirus drug screening, where the lead recognition rate improved significantly from 2.18% with SBVS alone to 16.44% with the combined approach [66].

#### 4.1.3 Cluster 3: Drug Design

This cluster includes the main keyword drug design. Artificial intelligence (AI) plays an important role in drug design and is used in target recognition, molecular screening, molecular structure prediction and optimization, and clinical trial prediction of drug design [67-71]. By combining big data analysis and AI algorithms, AI can accelerate the process of drug design and improve the success rate and accuracy of drug development [72-74].

AI enables the swift identification of drug targets through diverse data analysis, enhancing target discovery [75]. Freyhalt *et al*.'s RF model predicted drug-target binding with 84% accuracy, differentiating flexible, lipophilic membrane proteins from rigid, hydrophilic soluble ones [76]. Rifaioglu *et al*. DCNN-based DEEPScreen identified JAK protein as a novel target for cladribine [77]. Shao *et al*. GCN model DTI-HETA excels at predicting drug-target interactions from heterogeneous data [78].

AI is applied in molecular screening, which can predict the biological activity of new molecules based on known molecular structure and activity data, thereby screening candidate molecules with potential drug activity [79, 80]. This greatly reduces the number of molecules that require experimental verification and improves the efficiency of drug discovery [81]. Bian *et al*. developed a deep convolutional generative adversarial network (dcGAN) model for screening and designing novel compounds targeting cannabinoid receptors, which achieved good results [82].

AI predicts compound structures and properties by simulating intermolecular interactions, aiding in the optimization of molecular structures for drug design with improved efficacy and reduced side effects [83, 84]. CNN and RCNN methods have contributed to molecular structure prediction. Jiang *et al*. enhanced drug design accuracy using a CNN model, which provided a unique molecular structure image representation and a parallel CNN structure for predicting protein-ligand binding affinity [85]. The EResCNN model, integrating RCNN, XGBoost, RF, LightGBM, and extreme random tree, effectively predicts protein-protein interactions (PPIs) [86].

AI facilitates the analysis of historical clinical trial data, enabling the development of predictive models and decision-support tools for new drug trials [87, 88]. These applications can mitigate the risks and costs associated with clinical trials while hastening drug approval processes [88, 89]. ML methods, including SVM and RF, have been instrumental in clinical trial forecasting. ML classification models are valuable for identifying chemicals with potential Drug-induced immune thrombocytopenia (DITP) toxicity, offering a beneficial tool for drug development and clinical practice [90]. Accurate early prediction of drug-induced nephrotoxicity is pivotal for clinical trial success. SVM-based models have demonstrated efficacy in forecasting the nephrotoxicity of both chemical and traditional Chinese medicines [91]. Murali *et al*. introduced a Random Forest (RF) approach that leverages biological activity, physicochemical properties, and target-related features to reliably predict clinical trial outcomes [92].

#### 4.1.4 Cluster 4: Drug Discovery

This cluster includes the main keyword, drug discovery. Drug discovery is the process of discovering new candidate drugs [93]. Drug discovery is mainly divided into the following types of problems: Drug-target interactions (DTI), protein-ligand interactions, Drug response, Drug-combinations side effects and Drug-Drug similarity [94]. AI has played an important role in drug discovery. It has been applied in different stages of drug development [95, 96].

AI strategies are critical for early drug discovery, optimizing the Drug-target interactions (DTI) process to lower development costs and accelerate timelines [97, 98]. AI techniques, including CNNs, RNNs, and LDCNNs, enhance DTI prediction. Hu *et al*. demonstrated high CNN accuracy for predicting DTIs across various targets: 92% for enzymes, 90% for ion channels, 92% for GPCRs, and 90.7% for nuclear receptors [99]. Kavipriya *et al*. developed a semi-supervised RNN model for DTI prediction, showing strong experimental results [100]. Wang *et al*. introduced an LDCNN that predicts DTIs with less computational complexity than DeepConv, reducing neuron and filter counts by 50% with minimal performance loss [101].

Studying protein-ligand interactions is crucial for drug discovery. AI methods such as GCN, RNN, and CNN have played a role in the field of protein-large interactions. A GCN-based protein-ligand binding affinity prediction model, GraphBAR, can predict protein-ligand interactions at high performance and speed, making it a valuable tool for drug discovery [102]. The three-channel deep learning framework (SSGraphCPI) derived from RNN and GCN was used to predict compound protein interactions (CPI), achieving significant performance [103]. Using CNNs to identify peptide binding sites in predicting interactions between proteins and peptides can avoid expensive and time-consuming processes and achieve a sensitivity or recall rate of 67% (true positive rate) [104].

Drug response prediction plays a crucial role in drug discovery [105]. The use of well-trained AI models can accurately estimate drug response [106]. CNN and other AI methods have played a role in the field of drug response prediction [106, 107]. A drug response prediction algorithm combining 1D CNN and pathway network based on attention mechanism, with an average recognition accuracy of 84.6%, which is 4.5% higher than direct radio frequency methods [108]. A CNN-based joint graph sequence representation learning model for drug reaction prediction, called DGSDRP, can extract topological structure information from molecular graphs using graph convolutional networks (GCNs) [105]. The DL model is also widely used in the study of drug response characteristics and biomarkers based on cancer gene map data sets [106].

The use of drug combinations may result in side effects due to the interaction between drugs [109]. Using DL to predict drug-combination side effects can help reduce the likelihood of adverse reactions, save time and cost, and is superior to drug development and post-market monitoring processes [110, 111]. Graph convolutional neural network is used to predict the side effects of drug combinations. This method accurately predicts the side effects of multiple drugs, which is 69% higher than the baseline and achieves good performance [109].

Drug similarity features can be used to predict the association between drugs and diseases for drug discovery [112]. Kim Eunyoung, and others used ML algorithms to create predictive models using four types of drug similarity and three types of disease similarity to predict the unknown pharmacological effects of herbal compounds. The RF method showed the best performance (AUC=0.948). This model used clinical trial data to predict and validate new indications for 20 existing drugs and 31 herbal compounds [113]. Unsupervised ML methods can be used to define drug similarity and construct a weighted drug-drug similarity network based on drug-drug similarity relationships [114].

### 4.2 Research Frontiers

#### 4.2.1 Predictive Model (2020-2021)

In MC, the application of AI prediction models has become increasingly widespread [115, 116]. These models are mainly based on ML and DL techniques [117, 118]. Through training and learning a large amount of data, they can predict the biological activity of compounds, drug target interactions, and drug metabolism [71].

AI models streamline the identification of biologically active compounds, cutting experimental costs and time [119, 120]. John *et al*. used AI to evaluate COVID-19 antiviral compounds, with RF and XGBoost models showing 100% and 93.10% accuracy [121]. Malik *et al*. created an RF-based model to predict cholinesterase inhibitors, key for Alzheimer's research, identifying structural features like aromatic rings and amines that significantly affect activity [122].

AI models that predict drug-target interaction patterns and affinities are essential for understanding drug mechanisms and guiding drug design [123, 124]. Agyapong *et al*. developed an SVM-based model to predict the biological activity of small molecules with microtubule receptors, achieving an AUC of 87% and an accuracy of 93%, which aids in the development of potent anti-microtubule drugs [125]. Redkar *et al*. applied machine learning to study four protein types, predicting drug-target interactions with up to 96.3% accuracy, and also identified novel drug-target interactions [126].

Using AI models to predict the absorption, distribution, metabolism, and excretion (ADME) of drugs in the body, as well as the binding properties of drugs with plasma proteins. This helps to evaluate the safety and efficacy of drugs, guiding clinical medication and drug development [127]. Kumar, Kiran, and others used GCN algorithms to create a model that predicted 18 early ADME characteristics, demonstrating stronger predictive ability [115].

#### 4.2.2 Molecular Fingerprints (2021-2023)

In the field of MC, molecular fingerprints are an important tool for describing and comparing the structure and properties of molecules [128]. These fingerprints are composed of a series of numbers or binary codes that can capture key features of molecules, such as atomic type, bond type, and connectivity [129]. Molecular fingerprints play a crucial role in the combination of AI and MC. They provide key information about molecular structure for AI models, helping them make accurate predictions and accelerating the discovery and development process of new drugs, as well as optimizing and improving drugs [130, 131].

Molecular fingerprints combined with AI expedite the drug discovery and development process. AI techniques, including SVM, k-NN, FR, and RNN, have been instrumental in this domain. Sandhu *et al*. utilized SVM, k-NN, and RF algorithms to differentiate AChE inhibitors from non-inhibitors, with RF models achieving an accuracy of 85.38% for Alzheimer's drug target research [132]. In *de novo* drug design, Zhang *et al*. introduced a molecular deep generation RNN model using ligand-protein interaction fingerprints as constraints, showing a tendency to generate compounds with preserved binding patterns [133]. Huo *et al*. employed ECFP4 fingerprints for SVM and RF models and SMILES for a self-attention RNN model to analyze the impact of inhibitor side chains on EGFR activity, with model accuracies exceeding 0.87, contributing to cancer drug design [134].

Molecular fingerprints and AI are instrumental in drug optimization. Sun *et al*. developed SVM models using atomic classification descriptors and 3D fingerprints, achieving AUC-ROC values of 0.84 and 0.82, respectively, potentially guiding COVID-19 drug discovery [135]. Wang *et al*., in the context of rhinitis drug target CysLT1, constructed models with RF and DNN using CORINA descriptors and fingerprints, reaching a high accuracy of 93%, and identified substructures that enhance the biological activity of CysLT1 receptor antagonists [136].

#### 4.2.3 Scoring Function (2021–2023)

A scoring function is a mathematical or computational model used to evaluate or compare different options or results. In MC, scoring functions are commonly used in the drug design process to evaluate the binding affinity between potential drug molecules and biological targets [137, 138]. The scoring function developed based on artificial intelligence technology can correct the deviation of classical scoring functions and have higher predictive performance [139, 140]. The scoring function is very important in the field of AI-MC, as it can help accelerate the discovery process of new drugs, optimize drug properties, and assist in drug screening [141, 142].

Training AI models to forecast drug-target interactions, coupled with scoring functions to assess interaction strength, expedites new drug discovery [143]. Rayka *et al*. introduced the ET Score, an ML scoring function that significantly enhanced docking performance in drug design with a Pearson correlation coefficient of 0.827 and an RMSE of 1.332 [144]. Zaman *et al*. developed a DL model-based scoring function for protein structure prediction (PSP), refining protein specificity in the conformation search for informed decision-making [145]. Khashan *et al*. designed an ANN scoring function validated against PDBbind and Astex-85 databases, yielding satisfactory results [146].

MC can utilize AI algorithms and scoring functions to optimize the properties of existing drugs. By constructing a predictive model and combining it with experimental verification, precise regulation of drug molecular structure can be achieved [147]. Bjerrum *et al*. optimized drug design and improved efficiency and speed by using multi-objective scoring functions, dual loop reinforcement learning, and simplified molecular line-entry systems (SMILES) [148]. Wang *et al*. proposed a scoring function framework based on DL, which can identify key physical interactions in protein-ligand binding and optimize the ligand binding posture to the lowest energy conformation, with a success rate of 94.4% [149].

AI and scoring functions facilitate the development of computer-aided drug screening systems, which automatically analyze and screen potential drug molecules, reducing experimental demands [138, 150]. Wojcikowski *et al*. assessed eight scoring functions, finding that the RF function notably enhances virtual screening (VS) performance with improved affinity prediction, reflected in Pearson correlations of 0.56 and -0.18 [151]. Tran-Nguyen *et al*. compared CNNScore, a CNN-based scoring function, to classical and top-level structure fingerprint similarity scoring functions, concluding that CNNScore is superior for discovering new small molecule inhibitors, marking it as a promising structure-based VS method [152].

### 4.3 AI-MC Prospect

The application of AI-MC has shown enormous potential and prospects. Looking ahead to the future, with the continuous progress and innovation of technology, we can look forward to the development of multimodal and large language models (LLM) in the application of MC:

In the field of MC, multimodal techniques offer a more comprehensive and in-depth perspective for understanding and analyzing drug characteristics by integrating information from various sources, such as structural data and biological activity data of drug molecules [153-156]. The application of this technology is crucial to the drug design and optimization process, as it allows for more accurate predictions of drug properties and activities. For instance, the Multi-CycGT model proposed by Cao, Lu Jing, and colleagues, through multimodal analysis, is capable of predicting the one-dimensional and two-dimensional permeation properties of cyclic peptides with an average accuracy of 0.8206 and an area under the curve (AUC) of 0.8650. This model offers significant advantages for the design of active cyclic peptide drugs in the field of MC [157]. Additionally, the multimodal molecular transformer architecture designed by Zheng Shuangjia and colleagues, by integrating protein sequence information, is able to generate approximately 70% of molecules with improved biological activity. This rate is 1.9 times higher than that of other state-of-the-art deep learning methods, as well as rule-based and virtual screening approaches [158]. These cases demonstrate the potential of multimodal techniques in enhancing molecular biological activity and in the field of drug design, as well as their broad application prospects in MC research.

In the fields of MC and data analysis, Large Language Models (LLMs), such as ChatGPT, hold significant value due to their ability to generate relevant text. These models can assist researchers in designing arrays of chemical reactions, elucidating the mechanisms of drug action, and predicting potential side effects of drugs [159-162]. For example, Mahjour, Babak, and colleagues utilized ChatGPT to design an array of chemical reactions aimed at improving the yield of building block couplings. The model demonstrated exceptional performance in automated execution and analysis [163]. Furthermore, ChatGPT also successfully listed the most important drug classes used in the treatment of heart failure (HF) along with their mechanisms of action, showcasing its remarkable capability to provide comprehensive answers [164]. In an evaluation by Galido, Pearl Valentine, and their team, ChatGPT demonstrated the ability to accurately diagnose patients with treatment-resistant schizophrenia (TRS) and provided a detailed and highly informative compilation of potential drug side effects, offering an important tool for clinical reference [165]. These application cases highlight the immense potential of LLMs in drug design, understanding of disease mechanisms, and side effect prediction, opening new perspectives and tools for research in medicinal chemistry and biomedical sciences.

## CONCLUSION

In this research, we have comprehensively analyzed the application of AI in MC from 2001 to 2023, employing bibliometric and content analyses to map the field's evolution and identify key trends. The findings highlight four major research clusters: quantitative structure-activity relationship (QSAR) modeling, virtual screening, drug discovery, and drug design, each leveraging AI techniques such as deep learning, machine learning, and neural networks to enhance efficiency and accuracy. Emerging frontiers, including predictive models (2020-2021), molecular fingerprints (2021-2023), and scoring functions (2021-2023), underscore the transformative potential of AI in accelerating drug development and optimizing therapeutic outcomes. The integration of multimodal techniques and large language models (LLMs) further promises to revolutionize MC by enabling more precise drug design and analysis. Overall, this research provides a foundational framework for future AI-MC studies, emphasizing the critical role of AI in advancing drug discovery and development while reducing costs and time.

We identified some study limitations: The study relied on publications indexed in the Web of Science Core Collection, which may exclude relevant studies from other databases, potentially introducing selection bias; only English-language publications were included, omitting contributions from non-English journals, which may affect the global representativeness of findings.

## AUTHORS’ CONTRIBUTIONS

The authors confirm their contribution to the paper as follows: study conception and design: ZZ; data collection: TG; visualization: DX; validation: ML; draft manuscript: YW. All authors reviewed the results and approved the final version of the manuscript.

## Figures and Tables

**Fig. (1):**
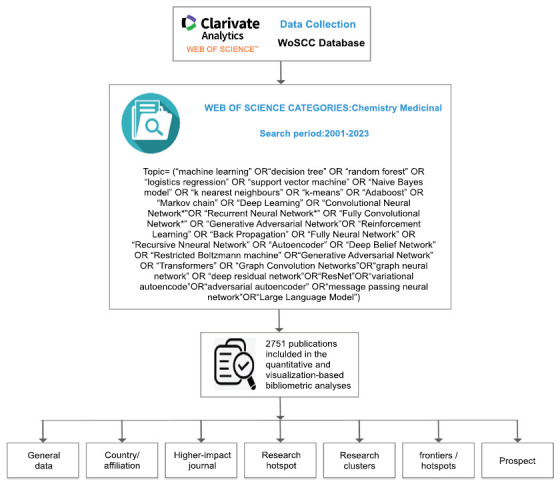
Study flow chart showing bibliometric analyses and selection criteria of AI-MC studies.

**Table 1:** Top ten countries/affiliations contributing to artificial intelligence-medicinal chemistry research.

**Rank**	**Country**	**Counts**	**Rank**	**Affiliations**	**Counts**
1	China (Asia)	582	1	Chinese Academy of Sciences	75
2	USA (North America)	572	2	Centre National De La Recherche Scientifique CNRS	66
3	India (Asia)	208	3	University of California System	62
4	Germany (Europe)	179	4	Astrazeneca	55
5	United Kingdom (Europe)	154	5	University of Cambridge	55
6	Japan (Asia)	115	6	United States Department of Energy DOE	49
7	France (Europe)	75	7	University of Bonn	47
8	Spain (Europe)	72	8	ETH Zurich	45
9	Brazil (South America)	69	9	Swiss Federal Institutes of Technology Domain	45
10	Iran (Asia)	66	10	ZHE Jiang University	40

**Table 2:** Top ten journals in artificial intelligence-medicinal chemistry research.

**Rank**	**Journal**	**Np**	**TC**	**h_index**	**g_index**	**PY_start**
1	Journal of Chemical Information And Modeling	612	18001	68	105	-
2	Molecular Informatics	142	2962	25	50	2010
3	Current Topics in Medicinal Chemistry	67	831	15	26	2006
4	Molecular Diversity	52	398	11	18	-
5	Journal of Medicinal Chemistry	48	1885	20	43	-
6	Current Medicinal Chemistry	42	614	11	18	2006
7	Pharmaceuticals	31	254	8	18	2018
8	Chemical Research in Toxicology	38	831	16	27	2001
9	Chemical Biology & Drug Design	35	342	9	18	2008
10	Journal of Pharmaceutical Sciences	30	324	10	18	2007

**Table 3:** The top ten cited documents on intelligence-medicinal chemistry studies.

**Rank**	**Journals**	**Study Title**	**Count**	**Findings**	**AI Algorithms**
1	J Med Chem	QSAR Modeling: Where Have You Been? Where Are You Going To?	104	The development and evolution of QSAR	-
2	J Chem Inf Model	Deep Neural Nets as a Method for Quantitative Structure-Activity Relationships [19]	100	Deep neural networks better predict quantitative structure-activity relationship datasets	Four-hidden-layer deep neural networks
3	J Chem Inf Model	Analyzing Learned Molecular Representations for Property Prediction [21]	90	Using graph convolutional models to predict molecular properties	Graph convolutional models
4	J Chem Inf Model	Protein-Ligand Scoring with Convolutional Neural Networks [20]	84	Convolutional neural networks for protein-ligand scoring	Convolutional neural networks
5	J Chem Inf Model	KDEEP: Protein-Ligand Absolute Binding Affinity Prediction *via* 3D-Convolutional Neural Networks	69	Using 3D convolutional neural networks to predict protein-ligand binding affinities	3D convolutional neural networks
6	J Chem Inf Model	Virtual Screening of Molecular Databases Using a Support Vector Machine	53	Applying a support vector machine (SVM) to virtual filtering is an effective method	Support vector machine
7	J Chem Inf Model	Deep Architectures and Deep Learning in Chemoinformatics: The Prediction of Aqueous Solubility for Drug-Like Molecules [22]	53	Using deep learning (DL) strategies to predict aqueous solubility of drug-like molecules	Recursive neural network
8	Mol Inform	Generative Recurrent Networks for *de novo* Drug Design [23]	53	Generative artificial intelligence models to design *de novo* drugs	Recursive neural network
9	J Chem Inf Model	GuacaMol: Benchmarking Models for *de novo* Molecular Design [24]	53	GuacaMol; an evaluation framework for *de novo* molecular design	Recurrent neural network
10	Mol Inform	Deep Learning in Drug Discovery [25]	49	Reviewing key DL approaches in drug discovery (DD)	Deep neural networks, restricted Boltzmann machine networks and convolutional networks

**Table 4:** The AI algorithm in MC.

**Application of AI**	**AI ** **Algorithms**	**Object**	**Data Sources**	**Dataset ** **Size**	**Accuracy%**	**AUC/MCC/** **R^2^/PCC**	**References**
**AI for QSAR**							
Prediction and search for inhibitors	RF, AdaBoost, ETR (optimal)	Establish a QSAR model to predict the inhibitory activity level of NS3, playing a significant advantage in the development, design, and screening of dengue fever	ChEMBL	845	ETR: 73	**AUC: ** ETR: 0.82	10.1080/1062936X.2020.1773534 [33]
Prediction and search for inhibitors	NB, SVM (optimal)	Predict chemical protein interactions, discover multitarget inhibitors, and provide a unique strategy for treating human immunodeficiency virus type-1 (HIV-1) and hepatitis C virus (HCV) coinfection	TTD ChEMBL	HIV-1: 11,006 HCV: 1,431	HIV-1: 93.18 HCV: 93.75	**AUC: ** HIV-1: 0.97, HCV: 0.96	10.3390/ijms20143572 [41]
Prediction and search for inhibitors	CNN	Predict the biological activity of inhibitors of anthrax and Discover potential anthrax-candidate drugs	AID 269502	10000	92.3	**R^2^: **0.79	10.4155/fmc-2023-0093 [42]
Predict and recognize the activity of anti-cancer molecules	RF, SVM, CNN	Build models for p53 inhibitor prediction, predict the activity of cancer proteins, and identify molecules with anticancer activity	ChEMBL ZINC Reaxys	10505	81	**MCC: **0.55	10.1016/j.bmc.2021.116530 [46]
Predict and recognize the activity of anti-cancer molecules	MLR, SVM	Discover anti-cancer lead	Chemdes	138	93.9	**R^2^: **0.481	10.2174/1573409919666221121141646 [41]
Predict and recognize the activity of anti-cancer molecules	MLR, ANN, SVM	Predicting the activity of anti-cancer drug	Literature	192	-	**R^2^: **>0.6	10.2174/1573406411309030014 [48]
**AI for Virtual Screening**							
SBVS	LR, GBT (optimal)	Improve the predictive ability of SBVS	CDK2, FXa, EGFR, HSP90	602	-	**AUC-ROC: **close to 0.5	10.1021/acs.jcim.1c00511 [57]
SBVS	Graph convolutional	Achieves better SBVS by successfully learning key features of protein-ligand interactions	PDBBindV2016, DUD-E, DEKOIS2.0, LIT-PCBA	8298	-	**AUC-ROC: **0.938	10.1021/acs.jmedchem.1c01830 [58]
LBVS	RF	Identified 15 specific hit compounds	ChEMBL, TIMBAL, DLiP,	620	RF-TI: 79 RF-PI: 99	**AUC-ROC: **RF-TI: 0.85, RF-PI:1 **MCC: ** RF-TI: 0.57, RF-PI: 0.98	10.1038/s41598-021-86616-1 [59]
LBVS	Bayesian learning	Online platform for LBVS	BindingDB, ChEMBL	-	-	**AUC: **0.86	10.1007/s11030-014-9545-3 [60]
LBVS	GMMs (optimal), Isolation Forests, ANN	Significantly improve the performance and efficiency of LBVS	DUD-E	38	-	**AUC-ROC:** 0.937 ± 0.037	10.3389/fphar.2019.01675 [61]
LBVS	DCNN	The model is highly effective in calculating molecular similarity and the performance of LBVS processes	MDDR	8568	98.97	-	10.1016/j.eswa.2022.117287 [62]
LBVS	GCN	Search for potential medicinal compounds from medicinal plants for the treatment of hemochromatosis	Phytochemical dataset	-	98.26	-	10.1109/ACCESS.2023.3338735 [63]
SBVS, LBVS	RF	Better predict binding affinity and potential future drug profiles	BindingDB, In-house xenobiotic, EDKB	1922	-	**R^2^: **0.71	10.1093/bioinformatics/btz538 [64]
SBVS, LBVS	RF	Predict the inhibitor activity of protein Monoamine Oxidase B (MAO-B), contributing to the development of drugs for the treatment of Parkinson's disease (PD)	Dukes Ethnobotanical Database, PubChem	108	-	-	10.2174/1573409915666190503113617 [65]
SBVS, LBVS	Integrating supervised learning	Coronavirus drug screening has significant advantages	USDA Phytochemical and Ethnobotanical Databases, CASF-2016 dataset	343/285	-	-	10.1080/07391102.2022.2112976 [66]
**AI for Drug Design**							
Target recognition	RF	Predict whether drugs bind to soluble or membrane proteins	DrugBank	1424	84	**AUC: **0.91	10.1002/minf.201400121 [76]
Target recognition	DCNN	Proposed a large-scale DTI prediction system	ChEMBL	704	87	**MCC: **0.74	10.1039/c9sc03414e [77]
Target recognition	GCN	Predicting the DTI from heterogeneous data sources, discovering novel DTIs	Own dataset Yamanishi dataset	2187	94	**AUC: **0.964	10.1093/bib/bbac109 [78]
Molecular screening	dcGAN	Screening and designing novel compounds targeting cannabinoid receptors	ChEMBL	-	-	**AUC: **0.944(max)	10.1021/acs.molpharmaceut.9b00500 [82]
Molecular structure prediction and optimization	CNN	Predicting protein-ligand binding affinity	PDBbind-v2007 PDBbind-v2013 PDBbind-v2016	PDBbind-v2007:1300 PDBbind-v2013:2959 PDBbind-v2016:4057	-	**PCC: **0.84265	10.1093/bib/bbab527 [85]
Molecular structure prediction and optimization	RCNN	Predicting protein-protein interactions	*Saccharomces cerevisiae* *Helicobacter pylori* Human-Y. pestis datasets	*Saccharomces cerevisiae*: 5594 *Helicobacter pylori*: 2916 Human-Y. pestis datasets:2067	-	**ACC: **98.61(max)	10.1016/j.compbiomed.2022.106471 [86]
Clinical trial prediction	SVM	Predicting the nephrotoxicity of chemical drugs and traditional Chinese medicine	SIDER, DrugBank, ChEMBL, TCM	1366	-	**ACC: **0.857 **AUC:** 0.915	10.1002/jat.4331 [91]
Clinical trial prediction	FR	Reliably and accurately predict clinical trial results	ChEMBL, DrugBank	-	93	**ACC:** 0.93 **AUC:** 0.83	10.1111/cbdd.14092 [92]
**AI for Drug Discovery**							
Drug-target interactions	RNN	Proposed a new semi-supervised DTI prediction method based on the optimal recurrent neural network model	Enzyme, Ion Channel, GPCR, Nuclear Receptor	Enzyme: 4035 Ion Channel: 1881 GPCR:953 Nuclear Receptor: 170	-	**AUC:** 0.9181	10.32604/iasc.2023.027670 [100]
Drug-target interactions	LDCNN	Proposed a new optical depth convolutional neural network (LDCNN) to predict DTI	Drugbank	25280	-	**AUC:** 0.8	10.1109/BIBM49941.2020.9313585 [101]
Protein-ligand interactions	GCN	Predict protein-ligand interactions at high performance and speed	PDBbind	64998	-	**R:** 0.754	10.1371/journal.pone.0249404 [102]
Protein-ligand interactions	RNN, GCN	Predict compound protein interactions	BindingDB	263583	-	**R^2^:** 0.66	10.3390/ijms23073780 [103]
Protein-ligand interactions	CNN	Using CNNs to identify peptide binding sites in predict interactions between proteins and peptides	BioLiP	1241	-	**AUC:** 0.73	10.1016/j.jtbi.2020.110278 [104]
Drug response prediction	CNN	A drug response prediction algorithm combining 1D CNN and pathway network based on attention mechanism	TCGA	130	84.6	-	10.1155/2022/8671348 [108]
Drug response prediction	DL	Estimate drug reactions in cancer genome map data	Encyclopedia	1156	80.88	-	10.1038/s41467-021-21997-5 [106]
Drug-combinations side effects	GCN	Predict the side effects of drug combinations	STITCH	8934	-	-	10.1093/bioinformatics/bty294 [109]
Drug-drug similarity	RF	Predict the unknown pharmacological effects of herbal compounds	DrugBank, OMIM)	1933	90.6	AUC: 0.948	10.1186/s12859-019-2811-8 [111]

**Abbreviations**: RF: random forest, AdaBoost: adaptive boosting, ETR: extremely randomized trees, NB: Na ve Bayes, SVM: support vector machine, MLR: multiple linear regression, ANN: artificial neural network, CNN: Convolutional Neural Network, LR: logistic regression, GBT: gradient boosting trees, GMMs: Gaussian Mixture Models, DCNN: Deep Convolutional Neural Network, GCN: Graph Convolutional Neural Networks, dcGAN: deep convolutional generative adversarial network, EResCNN: ensemble residual convolutional neural network, LDCNN: light deep convolutional neural network, ChEMBL: chemical and bioactivity database, TTD: Therapeutic Target Database, AID 269502: The dataset of small-molecule inhibitors of lethal anthrax factor, DUD-E: Decoys-Enhanced, MDDR: Drug Data Report Database, EDKB: Endocrine Disruptor Knowledge Base, SIDER: Side Effect Resource, R^2^: correlation coefficient.

## Data Availability

All the data and supporting information is provided within the article.

## LIST OF ABBREVIATIONS

3D: Three-dimensional
AI: Artificial Intelligence
AI-MC: MC AI Studies
ANN: Artificial Neural Network
BIMODAL: Bidirectional Molecule Design by Alternate Learning
BKs: Burst Keywords
CDK9: Cyclin-dependent Kinase 9
CNN: Convolutional Neural Network
DCNN: Deep Convolutional Neural Network
DD: Drug Discovery
DL: Deep Learning
DNN: Deep Neural Network
DPIs: Drug-protein Interactions
FBMD: Fragment-based Molecular Design
FCN: Fully Convolutional Network
FF: Force-field
GCN: Graph Convolutional Neural Networks
GNN: Graph Neural Network
LCS: Local Citation Score
LLM: Large Language Models
LSTM: Long Short-term Memory
MC: Medicinal Chemistry
MD: Molecular Docking
ML: Machine Learning
MTDNN: Multitask Deep Neural Network
NB: Naive Bayesian
QSAR: Quantitative Structure-activity Relationship
RF: Random Forest
RNN: Recurrent Neural Network
SBVS: Structure-based Virtual Screening
SMILES: Simplified Molecular Input Line Entry System
SVM: Support-vector Machine
TAFFI: Topology Automated FF Interactions
VS: Virtual Screening

## References

[1] Liang G.Q., Deng J., Wu C.X., Li W.J., Hu Y.F., Song Y.Q., Yin X.C., He Q., Xiao Y.C., Li G.B. (2023). Design, synthesis, and biological evaluation of boron-containing β-lactamase inhibitors: Closed-loop education experiences in an undergraduate medicinal chemistry course.. J. Chem. Educ..

[2] Fernandes J.P.S. (2018). The importance of medicinal chemistry knowledge in the clinical pharmacist’s education.. Am. J. Pharm. Educ..

[3] Hann M.M., Keserü G.M. (2012). Finding the sweet spot: The role of nature and nurture in medicinal chemistry.. Nat. Rev. Drug Discov..

[4] Gromek S., Balunas M. (2015). Natural products as exquisitely potent cytotoxic payloads for antibody- drug conjugates.. Curr. Top. Med. Chem..

[5] Fosso Wamba S., Bawack R.E., Guthrie C., Queiroz M.M., Carillo K.D.A. (2021). Are we preparing for a good AI society? A bibliometric review and research agenda.. Technol. Forecast. Soc. Change.

[6] Jiang F., Jiang Y., Zhi H., Dong Y., Li H., Ma S., Wang Y., Dong Q., Shen H., Wang Y. (2017). Artificial intelligence in healthcare: Past, present and future.. Stroke Vasc. Neurol..

[7] Lee S., Raza Shah S.A., Seok W., Moon J., Kim K., Raza Shah S.H. (2023). An optimal network-aware scheduling technique for distributed deep learning in distributed HPC platforms.. Electronics.

[8] Buccheri E., Dell’Aquila D., Russo M., Chiaramonte R., Vecchio M. (2024). Appendicular skeletal muscle mass in older adults can be estimated with a simple equation using a few zero-cost variables.. J. Geriatr. Phys. Ther..

[9] Dara S., Dhamercherla S., Jadav S.S., Babu C.H.M., Ahsan M.J. (2022). Machine learning in drug discovery: A review.. Artif. Intell. Rev..

[10] Vamathevan J., Clark D., Czodrowski P., Dunham I., Ferran E., Lee G., Li B., Madabhushi A., Shah P., Spitzer M., Zhao S. (2019). Applications of machine learning in drug discovery and development.. Nat. Rev. Drug Discov..

[11] Azar A.T., El-Metwally S.M. (2013). Decision tree classifiers for automated medical diagnosis.. Neural Comput. Appl..

[12] Buccheri E., Dell’Aquila D., Russo M., Chiaramonte R., Musumeci G., Vecchio M. (2023). Can artificial intelligence simplify the screening of muscle mass loss?. Heliyon.

[13] Batool M., Ahmad B., Choi S. (2019). A structure-based drug discovery paradigm.. Int. J. Mol. Sci..

[14] Yang X., Wang Y., Byrne R., Schneider G., Yang S. (2019). Concepts of artificial intelligence for computer-assisted drug discovery.. Chem. Rev..

[15] Merigó J.M., Gil-Lafuente A.M., Yager R.R. (2015). An overview of fuzzy research with bibliometric indicators.. Appl. Soft Comput..

[16] Mora L., Deakin M., Reid A. (2019). Combining co-citation clustering and text-based analysis to reveal the main development paths of smart cities.. Technol. Forecast. Soc. Change.

[17] Wang S., Zhou H., Zheng L., Zhu W., Zhu L., Feng D., Wei J., Chen G., Jin X., Yang H., Shi X., Lv X. (2021). Global trends in research of macrophages associated with acute lung injury over past 10 years: A bibliometric analysis.. Front. Immunol..

[18] Sun H.L., Bai W., Li X.H., Huang H., Cui X.L., Cheung T., Su Z.H., Yuan Z., Ng C.H., Xiang Y.T. (2022). Schizophrenia and inflammation research: A bibliometric analysis.. Front. Immunol..

[19] Ma J., Sheridan R.P., Liaw A., Dahl G.E., Svetnik V. (2015). Deep neural nets as a method for quantitative structure-activity relationships.. J. Chem. Inf. Model..

[20] Ragoza M., Hochuli J., Idrobo E., Sunseri J., Koes D.R. (2017). Protein–ligand scoring with convolutional neural networks.. J. Chem. Inf. Model..

[21] Yang K., Swanson K., Jin W.G., Coley C., Eiden P., Gao H., Guzman-Perez A., Hopper T., Kelley B., Mathea M., Palmer A., Settels V., Jaakkola T., Jensen K., Barzilay R. (2019). Analyzing learned molecular representations for property prediction.. J. Chem. Inf. Model..

[22] Lusci A., Pollastri G., Baldi P. (2013). Deep architectures and deep learning in chemoinformatics: The prediction of aqueous solubility for drug-like molecules.. J. Chem. Inf. Model..

[23] Gupta A., Müller A.T., Huisman B.J.H., Fuchs J.A., Schneider P., Schneider G. (2018). Generative recurrent networks for de novo drug design.. Mol. Inform..

[24] Brown N., Fiscato M., Segler M.H.S., Vaucher A.C. (2019). GuacaMol: Benchmarking Models for de novo molecular design.. J. Chem. Inf. Model..

[25] Gawehn E., Hiss J.A., Schneider G. (2016). Deep learning in drug discovery.. Mol. Inform..

[26] Ramsundar B., Liu B., Wu Z., Verras A., Tudor M., Sheridan R.P., Pande V. (2017). Is multitask deep learning practical for pharma?. J. Chem. Inf. Model..

[27] Jaeger S., Fulle S., Turk S. (2018). Mol2vec: Unsupervised machine learning approach with chemical intuition.. J. Chem. Inf. Model..

[28] Pereira J.C., Caffarena E.R., dos Santos C.N. (2016). Boosting docking-based virtual screening with deep learning.. J. Chem. Inf. Model..

[29] Cherkasov A., Muratov E.N., Fourches D., Varnek A., Baskin I.I., Cronin M., Dearden J., Gramatica P., Martin Y.C., Todeschini R., Consonni V., Kuz’min V.E., Cramer R., Benigni R., Yang C., Rathman J., Terfloth L., Gasteiger J., Richard A., Tropsha A. (2014). QSAR modeling: Where have you been? Where are you going to?. J. Med. Chem..

[30] Moriwaki H., Tian Y.S., Kawashita N., Takagi T. (2019). Three-dimensional classification structure–activity relationship analysis using convolutional neural network.. Chem. Pharm. Bull. (Tokyo).

[31] Andrade C.H., Pasqualoto K.F.M., Ferreira E.I., Hopfinger A.J. (2010). 4D-QSAR: Perspectives in drug design.. Molecules.

[32] Hameed R., Khan A., Khan S., Perveen S. (2019). Computational approaches towards kinases as attractive targets for anticancer drug discovery and development.. Anticancer. Agents Med. Chem..

[33] Kurniawan I., Rosalinda M., Ikhsan N. (2020). Implementation of ensemble methods on QSAR Study of NS3 inhibitor activity as anti-dengue agent.. SAR QSAR Environ. Res..

[34] Amoroso N., Quarto S., La Rocca M., Tangaro S., Monaco A., Bellotti R. (2023). An explainability artificial intelligence approach to brain connectivity in Alzheimer’s disease.. Front. Aging Neurosci..

[35] Yaprakdal F., Varol A.M. (2023). A multivariate time series analysis of electrical load forecasting based on a hybrid feature selection approach and explainable deep learning.. Appl. Sci..

[36] AlMohimeed A., Saad R.M.A., Mostafa S., El-Rashidy N.M., Farrag S., Gaballah A., Elaziz M.A., El-Sappagh S., Saleh H. (2023). Explainable artificial intelligence of multi-level stacking ensemble for detection of Alzheimer’s disease based on particle swarm optimization and the sub-scores of cognitive biomarkers.. IEEE Access.

[37] Kamal M.S., Chowdhury L., Nimmy S.F., Hasan Rafi T.H., Chae D-K. (2023). An Interpretable Framework for Identifying Cerebral Microbleeds and Alzheimer’s Disease Severity using Multimodal Data.. 2023 45th Annual International Conference of the IEEE Engineering in Medicine and Biology Society (EMBC).

[38] Calabrese V., Scapagnini G., Ravagna A., Bella R., Butterfield D.A., Calvani M., Pennisi G., Stella G.A.M. (2003). Disruption of thiol homeostasis and nitrosative stress in the cerebrospinal fluid of patients with active multiple sclerosis: Evidence for a protective role of acetylcarnitine.. Neurochem. Res..

[39] Calabrese V., Colombrita C., Guagliano E., Sapienza M., Ravagna A., Cardile V., Scapagnini G., Santoro A.M., Mangiameli A., Butterfield D.A., Stella A.M.G., Rizzarelli E. (2005). Protective effect of carnosine during nitrosative stress in astroglial cell cultures.. Neurochem. Res..

[40] Sun H., Wang J., Wu H., Lin S., Chen J., Wei J., Lv S., Xiong Y., Wei D.Q. (2023). A multimodal deep learning framework for predicting PPI-modulator interactions.. J. Chem. Inf. Model..

[41] Wei Y., Li W., Du T., Hong Z., Lin J. (2019). Targeting HIV/HCV coinfection using a machine learning-based multiple quantitative structure-activity relationships (Multiple QSAR) method.. Int. J. Mol. Sci..

[42] Kumari M., Subbarao N. (2023). Convolutional neural network-based quantitative structure-activity relationship and fingerprint analysis against inhibitors of anthrax lethal factor.. Future Med. Chem..

[43] Worachartcheewan A., Songtawee N., Siriwong S., Prachayasittikul S., Nantasenamat C., Prachayasittikul V. (2019). Rational design of colchicine derivatives as anti-HIV agents *via* QSAR and molecular docking.. Med. Chem..

[44] Deng Y., Liu Y., Tang S., Zhou C., Han X., Xiao W., Pastur-Romay L.A., Vazquez-Naya J.M., Loureiro J.P., Munteanu C.R., Tan Z. (2017). General machine learning model, review, and experimental-theoretic study of magnolol activity in enterotoxigenic induced oxidative stress.. Curr. Top. Med. Chem..

[45] Abbasi-Radmoghaddam Z., Riahi S., Gharaghani S., Mohammadi-Khanaposhtanai M. (2021). Design of potential anti-tumor PARP-1 inhibitors by QSAR and molecular modeling studies.. Mol. Divers..

[46] Patrício R.P.S., Videira P.A., Pereira F. (2022). A computer-aided drug design approach to discover tumour suppressor p53 protein activators for colorectal cancer therapy.. Bioorg. Med. Chem..

[47] Jayaprakash V., Saravanan T., Ravindran K., Prabha T., Selvaraj J., Jayapalan S., Chaitanya M.V.N.L., Sivakumar T. (2023). Relevance of machine learning to predict the inhibitory activity of small thiazole chemicals on estrogen receptor.. Curr. Computeraided Drug Des..

[48] Shayanfar A., Ghasemi S., Soltani S., Asadpour-Zeynali K., Doerksen R.J., Jouyban A. (2013). Quantitative structure-activity relationships of imidazole-containing farnesyltransferase inhibitors using different chemometric methods.. Med. Chem..

[49] Nayarisseri A., Khandelwal R., Tanwar P., Madhavi M., Sharma D., Thakur G., Speck-Planche A., Singh S.K. (2021). Artificial Intelligence, big data and machine learning approaches in precision medicine & drug discovery.. Curr. Drug Targets.

[50] Carpenter K.A., Cohen D.S., Jarrell J.T., Huang X. (2018). Deep learning and virtual drug screening.. Future Med. Chem..

[51] Kumar S.A., Ananda K.T.D., Beeraka N.M., Pujar G.V., Singh M., Narayana A.H.S., Bhagyalalitha M. (2022). Machine learning and deep learning in data-driven decision making of drug discovery and challenges in high-quality data acquisition in the pharmaceutical industry.. Future Med. Chem..

[52] Xiao T., Qi X., Chen Y., Jiang Y. (2018). Development of ligand‐based big data deep neural network models for virtual screening of large compound libraries.. Mol. Inform..

[53] Hirohara M., Saito Y., Koda Y., Sato K., Sakakibara Y. (2018). Convolutional neural network based on SMILES representation of compounds for detecting chemical motif.. BMC Bioinformatics.

[54] Torng W., Altman R.B. (2019). Graph convolutional neural networks for predicting drug-target interactions.. J. Chem. Inf. Model..

[55] Kumari M., Subbarao N. (2022). A hybrid resampling algorithms SMOTE and ENN based deep learning models for identification of Marburg virus inhibitors.. Future Med. Chem..

[56] Qin T., Zhu Z., Wang X.S., Xia J., Wu S. (2021). Computational representations of protein–ligand interfaces for structure-based virtual screening.. Expert Opin. Drug Discov..

[57] Ricci-Lopez J., Aguila S.A., Gilson M.K., Brizuela C.A. (2021). Improving structure-based virtual screening with ensemble docking and machine learning.. J. Chem. Inf. Model..

[58] Jiang D., Hsieh C.Y., Wu Z., Kang Y., Wang J., Wang E., Liao B., Shen C., Xu L., Wu J., Cao D., Hou T. (2021). InteractionGraphNet: A novel and efficient deep graph representation learning framework for accurate protein–ligand interaction predictions.. J. Med. Chem..

[59] Shimizu Y., Yonezawa T., Sakamoto J., Furuya T., Osawa M., Ikeda K. (2021). Identification of novel inhibitors of Keap1/Nrf2 by a promising method combining protein–protein interaction-oriented library and machine learning.. Sci. Rep..

[60] Zheng M., Liu Z., Yan X., Ding Q., Gu Q., Xu J. (2014). LBVS: An online platform for ligand-based virtual screening using publicly accessible databases.. Mol. Divers..

[61] Bonanno E., Ebejer J.P. (2020). Applying machine learning to ultrafast shape recognition in ligand-based virtual screening.. Front. Pharmacol..

[62] Berrhail F., Belhadef H., Haddad M. (2022). Deep convolutional neural network to improve the performances of screening process in LBVS.. Expert Syst. Appl..

[63] Ani R., Deepa O.S. (2023). Graph convolutional neural network-based virtual screening of phytochemicals and in-silico docking studies of drug compounds for hemochromatosis.. IEEE Access.

[64] Schneider M., Pons J.L., Bourguet W., Labesse G., Elofsson A. (2020). Towards accurate high-throughput ligand affinity prediction by exploiting structural ensembles, docking metrics and ligand similarity.. Bioinformatics.

[65] Mubashir N., Fatima R., Naeem S. (2020). Identification of novel phyto-chemicals from Ocimum basilicum for the treatment of Parkinson’s disease using in silico approach.. Curr. Computeraided Drug Des..

[66] Wang Z., Belecciu T., Eaves J., Reimers M., Bachmann M.H., Woldring D. (2023). Phytochemical drug discovery for COVID-19 using high-resolution computational docking and machine learning assisted binder prediction.. J. Biomol. Struct. Dyn..

[67] Sahu A., Mishra J., Kushwaha N. (2022). Artificial Intelligence (AI) in drugs and pharmaceuticals.. Comb. Chem. High Throughput Screen..

[68] Zhong F., Xing J., Li X., Liu X., Fu Z., Xiong Z., Lu D., Wu X., Zhao J., Tan X., Li F., Luo X., Li Z., Chen K., Zheng M., Jiang H. (2018). Artificial intelligence in drug design.. Sci. China Life Sci..

[69] Tripathi N., Goshisht M.K., Sahu S.K., Arora C. (2021). Applications of artificial intelligence to drug design and discovery in the big data era: A comprehensive review.. Mol. Divers..

[70] Piroozmand F., Mohammadipanah F., Sajedi H. (2020). Spectrum of deep learning algorithms in drug discovery.. Chem. Biol. Drug Des..

[71] Born J., Manica M. (2021). Trends in deep learning for property-driven drug design.. Curr. Med. Chem..

[72] Hudson I.L. (2021). Data integration using advances in machine learning in drug discovery and molecular biology.. Artif. Neural Networ.

[73] Lee J.W., Maria-Solano M.A., Vu T.N.L., Yoon S., Choi S. (2022). Big data and artificial intelligence (AI) methodologies for computer-aided drug design (CADD).. Biochem. Soc. Trans..

[74] Goel M., Aggarwal R., Sridharan B., Pal P.K., Priyakumar U.D. (2023). Efficient and enhanced sampling of drug‐like chemical space for virtual screening and molecular design using modern machine learning methods.. Wiley Interdiscip. Rev. Comput. Mol. Sci..

[75] Merchant J.P., Zhu K., Henrion M.Y.R., Zaidi S.S.A., Lau B., Moein S., Alamprese M.L., Pearse R.V., Bennett D.A., Ertekin-Taner N., Young-Pearse T.L., Chang R. (2023). Predictive network analysis identifies JMJD6 and other potential key drivers in Alzheimer’s disease.. Commun. Biol..

[76] Freyhult E., Gustafsson M.G., Strömbergsson H. (2015). A machine learning approach to explain drug selectivity to soluble and membrane protein targets.. Mol. Inform..

[77] Rifaioglu A.S., Nalbat E., Atalay V., Martin M.J., Cetin-Atalay R., Doğan T. (2020). Deepscreen: High performance drug–target interaction prediction with convolutional neural networks using 2-D structural compound representations.. Chem. Sci. (Camb.).

[78] Shao K., Zhang Y., Wen Y., Zhang Z., He S., Bo X. (2022). DTI-HETA: Prediction of drug–target interactions based on GCN and GAT on heterogeneous graph.. Brief. Bioinform..

[79] Jukič M., Bren U. (2022). Machine learning in antibacterial drug design.. Front. Pharmacol..

[80] Jing Y., Bian Y., Hu Z., Wang L., Xie X.Q.S. (2018). Deep learning for drug design: An artificial intelligence paradigm for drug discovery in the big data era.. AAPS J..

[81] D’Souza S., Kv P., Balaji S. (2022). Training recurrent neural networks as generative neural networks for molecular structures: How does it impact drug discovery?. Expert Opin. Drug Discov..

[82] Bian Y., Wang J., Jun J.J., Xie X.Q. (2019). Deep Convolutional Generative Adversarial Network (dcGAN) models for screening and design of small molecules targeting cannabinoid receptors.. Mol. Pharm..

[83] Peña-Guerrero J., Nguewa P.A., García-Sosa A.T. (2021). Machine learning, artificial intelligence, and data science breaking into drug design and neglected diseases.. Wiley Interdiscip. Rev. Comput. Mol. Sci..

[84] Mouchlis V.D., Afantitis A., Serra A., Fratello M., Papadiamantis A.G., Aidinis V., Lynch I., Greco D., Melagraki G. (2021). Advances in de novo drug design: From conventional to machine learning methods.. Int. J. Mol. Sci..

[85] Jiang P., Chi Y., Li X.S., Meng Z., Liu X., Hua X-S., Xia K. (2022). Molecular persistent spectral image (Mol-PSI) representation for machine learning models in drug design.. Brief. Bioinform..

[86] Gao H., Chen C., Li S., Wang C., Zhou W., Yu B. (2023). Prediction of protein-protein interactions based on ensemble residual convolutional neural network.. Comput. Biol. Med..

[87] Clayton E.A., Pujol T.A., McDonald J.F., Qiu P. (2020). Leveraging TCGA gene expression data to build predictive models for cancer drug response.. BMC Bioinformatics.

[88] Schperberg A.V., Boichard A., Tsigelny I.F., Richard S.B., Kurzrock R. (2020). Machine learning model to predict oncologic outcomes for drugs in randomized clinical trials.. Int. J. Cancer.

[89] Sharma A., Rani R. (2018). An integrated framework for identification of effective and synergistic anti-cancer drug combinations.. J. Bioinform. Comput. Biol..

[90] Wang B., Tan X., Guo J., Xiao T., Jiao Y., Zhao J., Wu J., Wang Y. (2022). Drug-induced immune thrombocytopenia toxicity prediction based on machine learning.. Pharmaceutics.

[91] Gong Y., Teng D., Wang Y., Gu Y., Wu Z., Li W., Tang Y., Liu G. (2022). In silico prediction of potential drug‐induced nephrotoxicity with machine learning methods.. J. Appl. Toxicol..

[92] Murali V., Muralidhar Y.P., Königs C., Nair M., Madhu S., Nedungadi P., Srinivasa G., Athri P. (2022). Predicting clinical trial outcomes using drug bioactivities through graph database integration and machine learning.. Chem. Biol. Drug Des..

[93] Sang S., Yang Z., Liu X., Wang L., Lin H., Wang J., Dumontier M. (2019). GrEDeL: A knowledge graph embedding based method for drug discovery from biomedical literatures.. IEEE Access.

[94] Askr H., Elgeldawi E., Aboul Ella H., Elshaier Y.A.M.M., Gomaa M.M., Hassanien A.E. (2023). Deep learning in drug discovery: An integrative review and future challenges.. Artif. Intell. Rev..

[95] Baskin I.I. (2020). The power of deep learning to ligand-based novel drug discovery.. Expert Opin. Drug Discov..

[96] Shi T., Yang Y., Huang S., Chen L., Kuang Z., Heng Y., Mei H. (2019). Molecular image-based convolutional neural network for the prediction of ADMET properties.. Chemom. Intell. Lab. Syst..

[97] Tian Q., Ding M., Yang H., Yue C., Zhong Y., Du Z., Liu D., Liu J., Deng Y. (2022). Predicting drug-target affinity based on recurrent neural networks and graph convolutional neural networks.. Comb. Chem. High Throughput Screen..

[98] Xu X., Xuan P., Zhang T., Chen B., Sheng N. (2022). Inferring drug-target interactions based on random walk and convolutional neural network.. IEEE/ACM Trans. Comput. Biol. Bioinformatics.

[99] Hu S., Xia D., Chen P., Wang B. (2018). Using novel convolutional neural networks architecture to predict drug-target interactions.. Intelligent Computing Theories and Application..

[100] Kavipriya G., Manjula D. (2023). Drug–target interaction prediction model using optimal recurrent neural network.. Intell. Aut Soft Comput..

[101] Wang S., Du Z., Ding M., Zhao R., Rodriguez-Paton A., Song T. LDCNN-DTI: A novel light deep convolutional neural network for drug-target interaction predictions.. 2020 IEEE International Conference on Bioinformatics and Biomedicine (BIBM).

[102] Son J., Kim D., Kim D. (2021). Development of a graph convolutional neural network model for efficient prediction of protein-ligand binding affinities.. PLoS One.

[103] Wang X., Liu J., Zhang C., Wang S. (2022). SSGraphCPI: A novel model for predicting compound-protein interactions based on deep learning.. Int. J. Mol. Sci..

[104] Wardah W., Dehzangi A., Taherzadeh G., Rashid M.A., Khan M.G.M., Tsunoda T., Sharma A. (2020). Predicting protein-peptide binding sites with a deep convolutional neural network.. J. Theor. Biol..

[105] Yan X., Liu Y., Zhang W. (2022). Deep graph and sequence representation learning for drug response prediction.. Artificial Neural Networks and Machine Learning – ICANN 2022..

[106] Jia P., Hu R., Pei G., Dai Y., Wang Y.Y., Zhao Z. (2021). Deep generative neural network for accurate drug response imputation.. Nat. Commun..

[107] Chen J., Zhang L. (2021). A survey and systematic assessment of computational methods for drug response prediction.. Brief. Bioinform..

[108] Mingxun Z., Zhigang M., Jingyi W., Ahmad S. (2022). Drug response prediction based on 1d convolutional neural network and attention mechanism.. Comput. Math. Methods Med..

[109] Zitnik M., Agrawal M., Leskovec J. (2018). Modeling polypharmacy side effects with graph convolutional networks.. Bioinformatics.

[110] Huang W., Li C., Ju Y., Gao Y. (2021). The next generation of machine learning in DDIs prediction.. Curr. Pharm. Des..

[111] Shim Y., Lee M., Kim P.J., Kim H.G. (2022). A novel approach to predicting the synergy of anti-cancer drug combinations using document-based feature extraction.. BMC Bioinformatics.

[112] Jiang H.J., You Z.H., Huang Y.A. (2019). Predicting drug−disease associations *via* sigmoid kernel-based convolutional neural networks.. J. Transl. Med..

[113] Kim E., Choi A., Nam H. (2019). Drug repositioning of herbal compounds *via* a machine-learning approach.. BMC Bioinformatics.

[114] Udrescu L., Bogdan P., Chiş A., Sîrbu I.O., Topîrceanu A., Văruţ R.M., Udrescu M. (2020). Uncovering new drug properties in target-based drug-drug similarity networks.. Pharmaceutics.

[115] Kumar K., Chupakhin V., Vos A., Morrison D., Rassokhin D., Dellwo M.J., McCormick K., Paternoster E., Ceulemans H., DesJarlais R.L. (2021). Development and implementation of an enterprise-wide predictive model for early absorption, distribution, metabolism and excretion properties.. Future Med. Chem..

[116] Siramshetty V.B., Nguyen D.T., Martinez N.J., Southall N.T., Simeonov A., Zakharov A.V. (2020). Critical assessment of artificial intelligence methods for prediction of hERG channel inhibition in the “Big Data” era.. J. Chem. Inf. Model..

[117] Korolev V., Mitrofanov A., Korotcov A., Tkachenko V. (2020). Graph convolutional neural networks as “General-Purpose” Property Predictors: The universality and limits of applicability.. J. Chem. Inf. Model..

[118] Hassan-Harrirou H., Zhang C., Lemmin T. (2020). RosENet: Improving binding affinity prediction by leveraging molecular mechanics energies with an ensemble of 3D convolutional neural networks.. J. Chem. Inf. Model..

[119] Fernandes P.O., Martins D.M., de Souza Bozzi A., Martins J.P.A., de Moraes A.H., Maltarollo V.G. (2021). Molecular insights on ABL kinase activation using tree-based machine learning models and molecular docking.. Mol. Divers..

[120] Safizadeh H., Simpkins S.W., Nelson J., Li S.C., Piotrowski J.S., Yoshimura M., Yashiroda Y., Hirano H., Osada H., Yoshida M., Boone C., Myers C.L. (2021). Improving measures of chemical structural similarity using machine learning on chemical–genetic interactions.. J. Chem. Inf. Model..

[121] John L., Soujanya Y., Mahanta H.J., Narahari Sastry G. (2022). Chemoinformatics and machine learning approaches for identifying antiviral compounds.. Mol. Inform..

[122] Malik A.A., Ojha S.C., Schaduangrat N., Nantasenamat C. (2022). ABCpred: A webserver for the discovery of acetyl- and butyryl-cholinesterase inhibitors.. Mol. Divers..

[123] Trapotsi M.A., Mervin L.H., Afzal A.M., Sturm N., Engkvist O., Barrett I.P., Bender A. (2021). Comparison of chemical structure and cell morphology information for multitask bioactivity predictions.. J. Chem. Inf. Model..

[124] Rodrigues C.H.M., Pires D.E.V., Ascher D.B. (2021). pdCSM-PPI: Using graph-based signatures to identify protein–protein interaction inhibitors.. J. Chem. Inf. Model..

[125] Agyapong O., Miller W.A., Wilson M.D., Kwofie S.K. (2022). Development of a proteochemometric-based support vector machine model for predicting bioactive molecules of tubulin receptors.. Mol. Divers..

[126] Redkar S., Mondal S., Joseph A., Hareesha K.S. (2020). A machine learning approach for drug‐target interaction prediction using wrapper feature selection and class balancing.. Mol. Inform..

[127] Grebner C., Matter H., Kofink D., Wenzel J., Schmidt F., Hessler G. (2021). Application of deep neural network models in drug discovery programs.. ChemMedChem.

[128] Zhao Y., Tian Y., Pang X., Li G., Shi S., Yan A. (2023). Classification of FLT3 inhibitors and SAR analysis by machine learning methods.. Mol. Divers..

[129] Nazarova A.L., Yang L., Liu K., Mishra A., Kalia R.K., Nomura K., Nakano A., Vashishta P., Rajak P. (2021). Dielectric polymer property prediction using recurrent neural networks with optimizations.. J. Chem. Inf. Model..

[130] Li R., Tian Y., Yang Z., Ji Y., Ding J., Yan A. (2023). Classification models and SAR analysis on HDAC1 inhibitors using machine learning methods.. Mol. Divers..

[131] Warszycki D., Struski Ł., Śmieja M., Kafel R., Kurczab R. (2021). Pharmacoprint: A combination of a pharmacophore fingerprint and artificial intelligence as a tool for computer-aided drug design.. J. Chem. Inf. Model..

[132] Sandhu H., Kumar R.N., Garg P. (2022). Machine learning-based modeling to predict inhibitors of acetylcholinesterase.. Mol. Divers..

[133] Zhang J., Chen H. (2022). De Novo molecule design using molecular generative models constrained by ligand–protein interactions.. J. Chem. Inf. Model..

[134] Huo D., Wang H., Qin Z., Tian Y., Yan A. (2022). Building 2D classification models and 3D Comsia models on small-molecule inhibitors of both wild-type and T790M/L858R double-mutant EGFR.. Mol. Divers..

[135] Sun H., Wang Y., Chen C.Z., Xu M., Guo H., Itkin M., Zheng W., Shen M. (2021). Identification of SARS-CoV-2 viral entry inhibitors using machine learning and cell-based pseudotyped particle assay.. Bioorg. Med. Chem..

[136] Wang H., Qin Z., Yan A. (2021). Classification models and SAR analysis on CysLT1 receptor antagonists using machine learning algorithms.. Mol. Divers..

[137] Scantlebury J., Vost L., Carbery A., Hadfield T.E., Turnbull O.M., Brown N., Chenthamarakshan V., Das P., Grosjean H., von Delft F., Deane C.M. (2023). A small step toward generalizability: Training a machine learning scoring function for structure-based virtual screening.. J. Chem. Inf. Model..

[138] Shan W., Li X., Yao H., Lin K. (2021). Convolutional neural network-based virtual screening.. Curr. Med. Chem..

[139] Veit-Acosta M., de Azevedo Junior W.F. (2021). The impact of crystallographic data for the development of machine learning models to predict protein-ligand binding affinity.. Curr. Med. Chem..

[140] Veit-Acosta M., de Azevedo Junior W.F. (2022). Computational prediction of binding affinity for CDK2-ligand complexes. A protein target for cancer drug discovery.. Curr. Med. Chem..

[141] Bitencourt-Ferreira G., de Azevedo Junior W.F. (2021). Electrostatic potential energy in protein-drug complexes.. Curr. Med. Chem..

[142] Sundin I., Voronov A., Xiao H., Papadopoulos K., Bjerrum E.J., Heinonen M., Patronov A., Kaski S., Engkvist O. (2022). Human-in-the-loop assisted de novo molecular design.. J. Cheminform..

[143] Rayka M., Firouzi R. (2023). GB‐score: Minimally designed machine learning scoring function based on distance‐weighted interatomic contact features.. Mol. Inform..

[144] Rayka M., Karimi-Jafari M.H., Firouzi R. (2021). ET‐score: Improving protein‐ligand binding affinity prediction based on distance‐weighted interatomic contact features using extremely randomized trees algorithm.. Mol. Inform..

[145] Zaman R., Newton M.A.H., Mataeimoghadam F., Sattar A. (2022). Constraint guided neighbor generation for protein structure prediction.. IEEE Access.

[146] Khashan R., Tropsha A., Zheng W. (2022). Data mining meets machine learning: A novel ANN-based multi‐body interaction docking scoring function (MBI-score) based on utilizing frequent geometric and chemical patterns of interfacial atoms in native protein‐ligand complexes.. Mol. Inform..

[147] Bieniek M.K., Cree B., Pirie R., Horton J.T., Tatum N.J., Cole D.J. (2022). An open-source molecular builder and free energy preparation workflow.. Commun. Chem..

[148] Bjerrum E.J., Margreitter C., Blaschke T., Kolarova S., de Castro R.L.R. (2023). Faster and more diverse de novo molecular optimization with double-loop reinforcement learning using augmented SMILES.. J. Comput. Aided Mol. Des..

[149] Wang Z., Zheng L., Wang S., Lin M., Wang Z., Kong A.W.K., Mu Y., Wei Y., Li W. (2023). A fully differentiable ligand pose optimization framework guided by deep learning and a traditional scoring function.. Brief. Bioinform..

[150] Zhang X., Shen C., Wang T., Kang Y., Li D., Pan P., Wang J., Wang G., Deng Y., Xu L., Cao D., Hou T., Wang Z. (2023). Topology-based and conformation-based decoys database: An unbiased online database for training and benchmarking machine-learning scoring functions.. J. Med. Chem..

[151] Wójcikowski M., Ballester P.J., Siedlecki P. (2017). Performance of machine-learning scoring functions in structure-based virtual screening.. Sci. Rep..

[152] Tran-Nguyen V.K., Simeon S., Junaid M., Ballester P.J. (2022). Structure-based virtual screening for PDL1 dimerizers: Evaluating generic scoring functions.. Curr. Res. Struct. Biol..

[153] Marivani I., Tsiligianni E., Cornelis B., Deligiannis N. (2022). Designing CNNs for multimodal image restoration and fusion *via* unfolding the method of multipliers.. IEEE Trans. Circ. Syst. Video Tech..

[154] Razzaghi P., Abbasi K., Shirazi M., Rashidi S. (2022). Multimodal brain tumor detection using multimodal deep transfer learning.. Appl. Soft Comput..

[155] Yuzhakova D.V., Lermontova S.A., Grigoryev I.S., Muravieva M.S., Gavrina A.I., Shirmanova M.V., Balalaeva I.V., Klapshina L.G., Zagaynova E.V. (2017). *In vivo* multimodal tumor imaging and photodynamic therapy with novel theranostic agents based on the porphyrazine framework-chelated gadolinium (III) cation.. Biochim. Biophys. Acta, Gen. Subj..

[156] Race A.M., Sutton D., Hamm G., Maglennon G., Morton J.P., Strittmatter N., Campbell A., Sansom O.J., Wang Y., Barry S.T., Takáts Z., Goodwin R.J.A., Bunch J. (2021). Deep learning-based annotation transfer between molecular imaging modalities: An automated workflow for multimodal data integration.. Anal. Chem..

[157] Cao L., Xu Z., Shang T., Zhang C., Wu X., Wu Y., Zhai S., Zhan Z., Duan H. (2024). Multi_CycGT: A deep learning-based multimodal model for predicting the membrane permeability of cyclic peptides.. J. Med. Chem..

[158] Zheng S., Lei Z., Ai H., Chen H., Deng D., Yang Y. (2021). Deep scaffold hopping with multimodal transformer neural networks.. J. Cheminform..

[159] Pradhan T., Gupta O., Chawla G. (2024). The future of chatGPT in medicinal chemistry: Harnessing AI for accelerated drug discovery.. ChemistrySelect.

[160] Jinsong S., Qifeng J., Xing C., Hao Y., Wang L. (2024). Molecular fragmentation as a crucial step in the AI-based drug development pathway.. Commun. Chem..

[161] Telenti A., Auli M., Hie B.L., Maher C., Saria S., Ioannidis J.P.A. (2024). Large language models for science and medicine.. Eur. J. Clin. Invest..

[162] Wu Z., Chen J., Li Y., Deng Y., Zhao H., Hsieh C.Y., Hou T. (2023). From black boxes to actionable insights: A perspective on explainable artificial intelligence for scientific discovery.. J. Chem. Inf. Model..

[163] Mahjour B., Hoffstadt J., Cernak T. (2023). Designing chemical reaction arrays using phactor and ChatGPT.. Org. Process Res. Dev..

[164] Dimitriadis F., Alkagiet S., Tsigkriki L., Kleitsioti P., Sidiropoulos G., Efstratiou D., Askalidi T., Tsaousidis A., Siarkos M., Giannakopoulou P., Mavrogianni A.D., Zarifis J., Koulaouzidis G. (2024). ChatGPT and patients with heart failure.. Angiology.

[165] Galido P.V., Butala S., Chakerian M., Agustines D. (2023). A case study demonstrating applications of chatgpt in the clinical management of treatment-resistant schizophrenia.. Cureus.

